# In Vivo Radioprotective Activity of Cell-Permeable Bifunctional Antioxidant Enzyme GST-TAT-SOD against Whole-Body Ionizing Irradiation in Mice

**DOI:** 10.1155/2017/2689051

**Published:** 2017-07-19

**Authors:** Jianru Pan, Huocong He, Ying Su, Guangjin Zheng, Junxin Wu, Shutao Liu, Pingfan Rao

**Affiliations:** ^1^College of Biological Science and Engineering, Fuzhou University, No. 2 Xue Yuan Road, University Town, Fuzhou, Fujian 350108, China; ^2^Laboratory of Radiation Oncology and Radiobiology, Fujian Cancer Hospital & Fujian Medical University Cancer Hospital, Fujian Key Laboratory of Tumor Translational Cancer Medicine, The National Clinical Key Specialty Construction Program of China, No. 420 Fuma Road, Fuzhou 350014, China; ^3^Food Nutrition Sciences Centre, Zhejiang Gongshang University, Room 407, No. 1 Laboratory Building, No. 149 Jiaogong Road, Xihu District, Hangzhou 310012, China

## Abstract

GST-TAT-SOD was the fusion of superoxide dismutase (SOD), cell-permeable peptide TAT, and glutathione-S-transferase (GST). It was proved to be a potential selective radioprotector in vitro in our previous work. This study evaluated the in vivo radioprotective activity of GST-TAT-SOD against whole-body irradiation. We demonstrated that intraperitoneal injection of 0.5 ml GST-TAT-SOD (2 kU/ml) 2 h before the 6 Gy whole-body irradiation in mice almost completely prevented the splenic damage. It could significantly enhance the splenic antioxidant activity which kept the number of splenic white pulp and consequently resisted the shrinkage of the spleen. Moreover, the thymus index, hepatic antioxidant activity, and white blood cell (WBC) count of peripheral blood in irradiated mice pretreated with GST-TAT-SOD also remarkably increased. Although the treated and untreated irradiated mice showed no significant difference in the growth rate of animal body weight at 7 days postirradiation, the highest growth rate of body weight was observed in the GST-TAT-SOD-pretreated group. Furthermore, GST-TAT-SOD pretreatment increased resistance against 8 Gy whole-body irradiation and enhanced 30 d survival. The overall effect of GST-TAT-SOD seemed to be a bit more powerful than that of amifostine. In conclusion, GST-TAT-SOD would be a safe and potentially promising radioprotector.

## 1. Introduction

Approximately 50% of all cancer patients received radiation therapy during their course of illness. Radiation therapy is known to kill cancer cells effectively. However, it can also damage healthy cells in addition to cancer cells, leading to side effects termed radiation sickness. Radiation therapy side effects are mainly induced by the reactive oxygen species (ROS) such as superoxide, hydroxyl radicals, and hydrogen peroxide produced through the radiolysis of the water [1]. These free radicals react with critical cellular macromolecules resulting in cell dysfunction and death, depletion of stem cell pools, and organ system dysfunction [2].

The elimination of the free radical species from the cell environment can inhibit the side effects induced by irradiation [3]. Amifostine, a radioprotector in use clinically, can freely diffuse into cells and can act as a free radical scavenger through dephosphorylation [4]. It is the only cytoprotective agent specifically approved by the FDA as a radioprotector. However, it had low potency and poor bioavailability due to the stoichiometric nature of its action [5]. Moreover, side effects of amifostine such as fever, rash, severe nausea, allergy, and acute hypotension have been reported increasingly [6–8]. There is a continued need for the development of a nontoxic and efficient radioprotector.

As the superoxide radicals produced by ionizing radiation are highly reactive and potentially damaging to cells, the enzyme superoxide dismutase (SOD) should be radioprotective. Many studies have supported the hypothesis through transgenic experiments [9–13]. However, the direct administration of wild SOD was inefficient as it is too large to enter into cells freely.

A cell-penetrating peptide derived from the HIV-1 Tat protein transduction domain TAT (YGRKKRRQRRR) can carry larger molecules across cellular membranes. It is useful in delivering biologically active cargoes in both in vitro and in vivo models [14–18]. The cell-permeable recombinant protein SOD-TAT constructed with the fusion of hCuZn-SOD (SOD1) and TAT was remarkably effective in preventing the radio-induced skin or lung injury in vivo [19–22].

However, superoxide radicals were not the only harmful reactive chemical species produced by ionizing radiation. Hence, a cell-permeable bifunctional antioxidant enzyme fused with glutathione-S-transferase (GST) and cell-permeable SOD was constructed and named GST-TAT-SOD [23]. GST is an enzyme that aids in detoxification by speeding up the linking of toxic compounds with glutathione (GSH), thus forming a less reactive substance. The cell-permeable bifunctional antioxidant enzyme had a remarkable protective effect on irradiated normal liver cells and a minimal effect on irradiated hepatoma cells. It is superior to SOD-TAT and amifostine in an in vitro experiment [23].

The aim of this study was to evaluate the radioprotective effects of the cell-permeable bifunctional GST-TAT-SOD on the whole-body irradiated mice compared with those of amifostine.

## 2. Materials and Methods

### 2.1. Enzyme and Chemicals

*E. coli* strains with the recombinant plasmid of GST-TAT-SOD were obtained from the Institute of Biotechnology, Fuzhou University (Fujian, China). Amifostine was purchased from Meiluo Yinhe Pharmacy Co. Ltd. (Hunan, China). Malondialdehyde (MDA), superoxide dismutase (SOD), and glutathione-S-transferase (GST) reagent kits were purchased from Nanjing Jiancheng Bioengineering Co. Ltd. (Jiangsu, China). The Micro BCA™ Protein Assay Kit was purchased from Thermo Scientific (USA). All other chemicals were of analytical purity.

### 2.2. Mice

Male Swiss albino mice (Fujian Medical University) weighing 18–22 g each were used at 6–8 weeks of age for these experiments. All mice were housed in an animal room at 22°C in a 12 h light/12 h dark cycle. All mice were given a standard chow diet and water ad libitum. Animal welfare and experimental procedures were carried out in accordance with the Guide for the Care and Use of Laboratory Animals (Ministry of Science and Technology of China, 2006) and were approved by the Review Committee for the Use of Human or Animal Subjects of the Institute of Biotechnology, Fuzhou University.

### 2.3. Preparation of GST-TAT-SOD

GST-TAT-SOD was prepared according to the method of our previous work [23]. The concentration and SOD activity and GST activity of the purified protein were determined by the BCA protein assay kit (Thermo, USA) and SOD and GST reagent kits (Jiangsu, China), respectively. The SOD and GST activity of purified GST-TAT-SOD was 2476 and 766 U/ml, respectively. The purified protein was concentrated and dialyzed for subsequent experiments. The activity of GST-TAT-SOD mentioned from now on referred to its SOD activity.

### 2.4. Radiation

Mice were placed in well-ventilated Perspex boxes of dimensions 23.5 cm × 23.5 cm × 3.5 cm, partitioned into 3 cm × 3 cm × 11 cm cells for individual animals. They were exposed to whole-body irradiation from X-ray generated by a LINAC (IEC 61217) with a nominal potential of 6 MV and a dose rate of 300 UM/min with a source-to-surface distance of 100 cm. The radiation dose to the mice was selected according to an unpublished preliminary study. Our results showed that the radiation dose of 6 Gy causes acute damages of hematopoietic and immune systems, but it was not fatal within 30 days. However, 8 Gy whole-body irradiation caused 100% mortality within 18 days. So we chose two radiation doses (6 Gy and 8 Gy) to assay the protective effect of GST-TAT-SOD on nonlethal irradiation damage and the survival rate from lethal irradiation damage, respectively.

### 2.5. Treatment of Mice

To establish an acute whole-body irradiation damage model, the mice were randomly divided into 6 groups (*n* = 8/group). The control group (CON) was unirradiated and untreated. The GST-TAT-SOD group was unirradiated and single intraperitoneally injected with a dose of 2000 U/ml (0.5 ml). The positive control group was treated with 200 mg/kg amifostine (XRT + AMFT) dissolved in saline and administered intraperitoneally at 30 min before irradiation while the negative control group (XRT) remained untreated. The XRT + GST-TAT-SOD group was treated with IP administration at a dose of 2000 U/ml (0.5 ml) GST-TAT-SOD 2 h before irradiation. The time point and dose of the GST-TAT-SOD protein were determined according to the results of previous in vitro work [23] and an unpublished preliminary experiment exposed to whole-body irradiation. The mice were irradiated at a dose of 6 Gy at room temperature. Seven days after irradiation, all animals were sacrificed by cervical dislocation.

### 2.6. Growth Rate of Body Weight

Each mouse was weighed before irradiation and 7 days after irradiation, and the growth rate of the body weight was calculated.

### 2.7. White Blood Cells (WBC), Spleen Index, and Thymus Index

Blood was collected from the orbital artery 7 days after irradiation, and the WBC were counted using a hemocytometer after the blood was diluted. Then, the spleen and thymus were removed, and the spleen index and thymus index were calculated (spleen or thymus weight/body weight × 100).

### 2.8. Histopathological Study

In the histological studies, portions of the spleen were fixed with formalin, dehydrated in graded (50–100%) alcohol, and embedded in paraffin. Thin sections (4-5 *μ*m) were cut and stained with hematoxylin and eosin (HE) stain. They were analyzed by a light microscope (Olympus BX31), and images were captured with a digital charge-coupled device camera (Olympus DP72) and a PC for data acquisition and analysis.

### 2.9. Measurement of SOD, MDA, and GST Activity

The spleen and liver dissected out were weighed, and 10% homogenate was prepared with ice-cold saline using a homogenizer (IKA T10 basic, USA). The activity of SOD (U/mg protein), MDA (nmol/mg protein), and GST (U/mg protein) was determined spectrophotometrically using their corresponding diagnostic reagent kits (Nanjing Jiancheng Bioengineering) according to the manufacturer's instructions. The protein contents of 10% homogenate were determined by using the BCA protein assay kit (Thermo, USA).

### 2.10. Survival Studies

To establish a lethal whole-body irradiation model, 3 groups of mice (*n* = 10/group) were used. The positive control group was treated with 200 mg/kg amifostine (XRT + AMFT) dissolved in saline and administered IP at 30 min before irradiation while the negative control group remained untreated. GST-TAT-SOD (XRT + GST-TAT-SOD) was used for IP administration at a dose of 2000 U/ml (0.5 ml) to animals before irradiation. Animals received a dose of 8 Gy followed by 30 days of observation. The number of surviving mice was recorded daily up to 30 days postirradiation, and the data were expressed as percentage survival.

### 2.11. Statistical Analyses

Statistical analysis of all data was performed using Excel. The results are reported as means ± SE or SEM. The *P* values were determined using the Student two-tailed *t*-test, and *P* < 0.05 or *P* < 0.01 was considered statistically significant.

## 3. Result

### 3.1. Growth Rate of Body Weight

**Figure 1:**
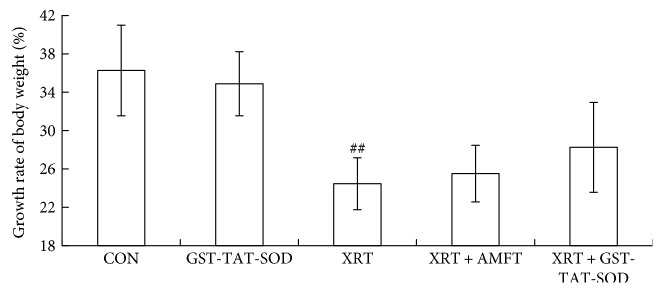
The effect of GST-TAT-SOD on the growth rate of body weight over 7 days after 6 Gy whole-body irradiation. Values are expressed as means ± SD (*n* = 8; compared with control group, ^#^^#^*P* < 0.01).

The growth of the animals was assessed by monitoring body weights of the animals 7 days after whole-body irradiation at a dose of 6 Gy, as shown in Figure 1. The growth rate of body weight was increasing in all groups. There was no notable difference between the GST-TAT-SOD group and CON group. A significantly decreased growth rate was observed in the XRT group compared with the CON group (*P* < 0.05). Administration of amifostine or GST-TAT-SOD seemed to effect on maintaining irradiated mice's body weight, and the latter was more efficient than the former. However, there is no statistical significance between the treated groups and XRT group.

### 3.2. WBC

**Figure 2:**
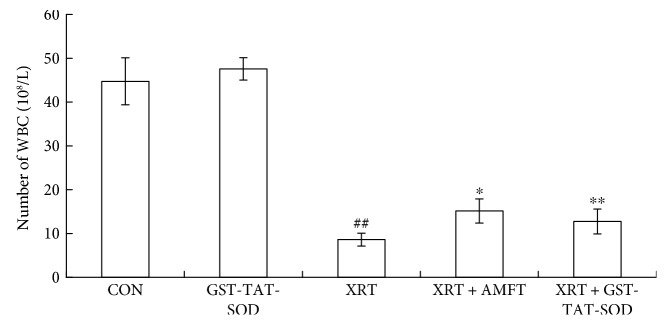
Effects of GST-TAT-SOD on WBC in the peripheral blood of mice exposed to 6 Gy whole-body irradiation. Blood was collected from the orbital artery 7 days after irradiation, and the WBC were counted using a hemocytometer (*n* = 8; compared with control group, ^#^^#^*P* < 0.01, compared with XRT group, ^∗^*P* < 0.05, ^∗∗^*P* < 0.01).

In the present study, alterations in the WBC count were found in all groups (Figure 2). No remarkable difference in the number of WBC was observed between the GST-TAT-SOD group and CON group while that in the XRT group was significantly (*P* < 0.05) lower than that in the CON group. All of the pretreatments (*P* < 0.05 and *P* < 0.01, resp.) could significantly enhance the recovery of the parameter in irradiated mice.

### 3.3. Thymus Index and Spleen Index

**Figure 3:**
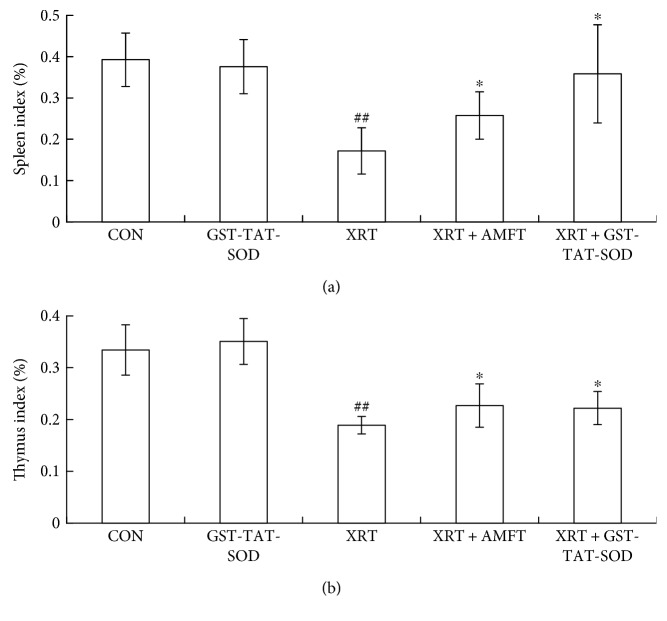
Effect of GST-TAT-SOD on the spleen index (a) and thymus index (b) in mice exposed to 6 Gy whole-body irradiation. Animals were sacrificed 7 days after irradiation. Then, the spleen and thymus were removed, and the spleen index and thymus index were calculated (spleen or thymus weight/body weight × 100) (*n* = 8; compared with control group, ^#^^#^*P* < 0.01, compared with XRT group, ^∗^*P* < 0.05).

The results of the immune organ study were presented in Figure 3. As shown in Figure 3, the spleen index and thymus index in the CON group versus GST-TAT-SOD groups were not remarkably different, but a significant reduction (*P* < 0.01) in those indices was observed in radiation-alone group. However, two indices of pretreatment with amifostine or GST-TAT-SOD were significantly increased (*P* < 0.05), especially the latter, which maintained the spleen index close to that in unirradiated normal mice.

### 3.4. Histopathological Study

**Figure 4:**
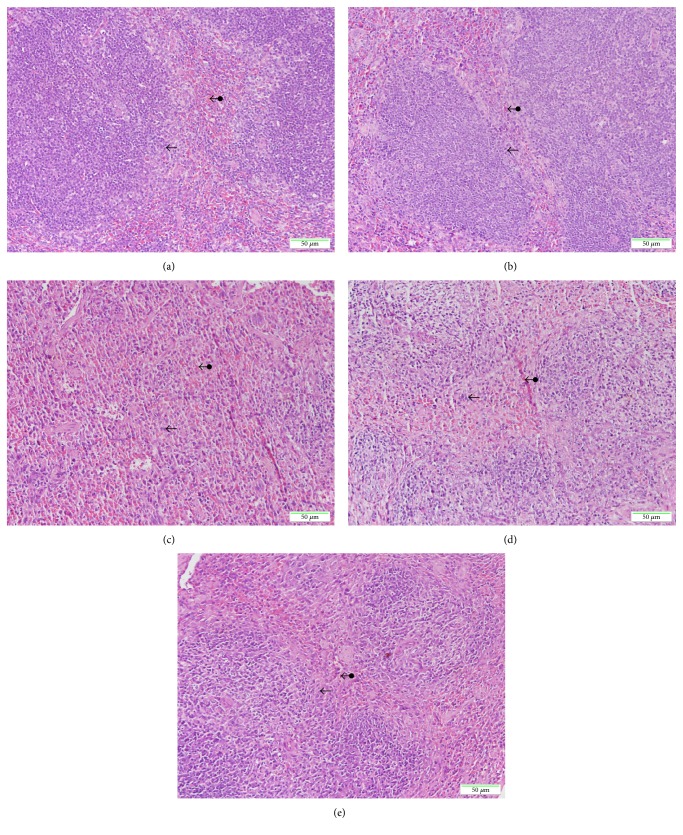
Histopathological demonstration of the spleen using a light microscope. Mice were sacrificed 7 days after 6 Gy whole-body irradiation. Portions of spleen tissues were fixed, embedded, cut into sections, and stained with hematoxylin and eosin for observation using a light microscope. Micrograph of splenic tissue showing the red pulp (double-headed arrows) and white pulp (single-headed arrows). CON group (a); GST-TAT-SOD group (b); XRT group (c); XRT + AMFT group (d); XRT + GST-TAT-SOD group (e).

Histologic analysis revealed that exposure to X-ray irradiation resulted in remarkable changes in the spleens of mice at the 7th day postirradiation, as shown in Figure 4. In the spleen, there was no difference between the unirradiated CON and GST-TAT-SOD groups (Figures 4(a) and 4(b)), which both presented the well-defined red pulp and abundant white pulp. Irradiation appeared to cause the disappearance of vast numbers of white pulp, and the boundaries of the white pulp and red pulp were vague (Figure 4(c)). Amifostine pretreatment seemed to maintain the amount of white pulp to some degree (Figure 4(d)). When mice were treated with GST-TAT-SOD before irradiation, the histopathological lesions were not observed in the splenic tissues and the spleens appeared similar to the control group, showing a remarkably increase in the white pulp (Figure 4(e)).

### 3.5. Measurement of SOD, MDA, and GST Activity

No significant difference was found between the GST-TAT-SOD group and control group with regard to splenic antioxidant indices (Table 1). Whole-body radiation significantly (*P* < 0.05) increased the MDA level in the spleen of mice exposed to 6 Gy irradiation. Compared with the radiation-alone group, the splenic MDA levels significantly reduced by 35.4% (*P* < 0.05) and 29.0% (*P* < 0.05) in the radiation plus amifostine or radiation plus GST-TAT-SOD groups, respectively. A significant (*P* < 0.01) downward trend in SOD activity was observed in the spleen of the radiation-alone group at 7 d postirradiation (Table 1) while the pretreatment of either amifostine or GST-TAT-SOD significantly elevated SOD activity by 68.8% (*P* < 0.05) and 90.2%, respectively. The splenic GST activity of mice was kept steady, and the differences were not significant among each group. The situation of the hepatic antioxidant indices is quite similar to that of the spleen as shown in Table 2. However, we found that GST-TAT-SOD pretreatment obviously is more efficient in reducing the MDA level in irradiated mice compared with amifostine. Moreover, only GST-TAT-SOD pretreatment could remarkably enhance irradiated animal's hepatic SOD activity back to normal levels (*P* < 0.01).

### 3.6. Survival Studies

**Figure 5:**
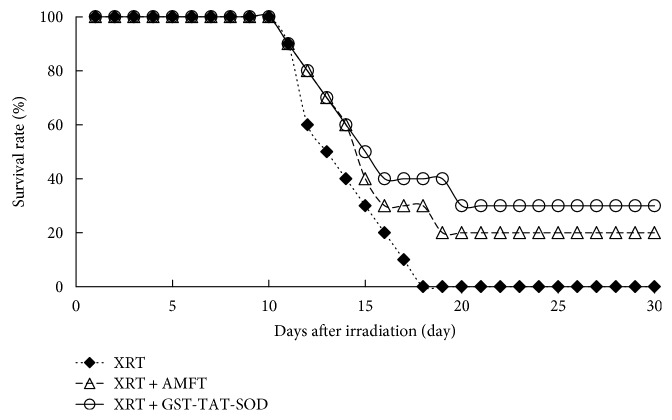
Thirty-day survival of mice exposed to 8 Gy whole-body irradiation. The lines represent animal survival rates for 10 mice per treatment group.

Mortality was seen in the range of 10–100% on days 11–18 after WBI (Figure 5). However, the pretreatment of mice with amifostine or GST-TAT-SOD caused a remarkable improvement in their survival. In the amifostine-treated groups, 30% mice survived within 18 days and 20% mice survived after 30 days. When pretreated with GST-TAT-SOD, 40% mice survived within 18 days and 30% mice survived after 30 days.

## 4. Discussion

Acute exposure to ionizing radiation can have fatal effects on the hematopoietic and immune systems. WBCs appeared to be the most sensitive indicator of the hematopoietic system to irradiation among the types of blood cells evaluated [24]. Lymphocytes are also extremely radiosensitive and have been suggested as a biological dosimeter [25, 26]. In addition to the loss from tile circulation, morphological changes rapidly appear in the lymphoid tissues, such as those in the spleen and thymus, which quickly decrease in size.

In this study, a significant deficit in the WBC of peripheral blood and the shrunken spleen and thymus were observed in mice of the X-ray irradiation-alone group (Figures 2 and 3). Both GST-TAT-SOD and amifostine pretreatments showed remarkable recovery of the above irradiation-induced injury (Figures 2 and 3). Compared with amifostine, GST-TAT-SOD was more efficient on protecting the spleen as its pretreatment could maintain the spleen index close to that in normal mice (Figure 3(a)). The splenic white pulp produces and grows immune cells as well as blood cells. Its quantity was significantly decreased in irradiated mice (Figure 4(a)). When mice were pretreated with GST-TAT-SOD, the numbers of splenic white pulp appeared similar to those of the control group and the splenic tissues showed a remarkable recovery (Figure 4(e)).

Most of the radiation-induced damage is caused by the formation of free radicals resulting from the radiolysis of water [27]. Reactive oxygen species-mediated cascading chain reactions and redox imbalances have been well documented in radiation toxicity studies. MDA is generated by free radical attack on cell membrane phospholipids and circulating lipids and acts as a sensitive biomarker for oxidative stress that occurs as part of the pathogenesis of various diseases [28]. Superoxide is considered to be the most harmful ROS due to its high reactivity. The endogenous SOD is the only antioxidant enzyme responsible for the deactivation of superoxide ion in cells. It catalyzes the dismutation of the superoxide ion (O^2−^) and converts it to H_2_O_2_ [29].

Many transgenic experiments of SOD have provided the proofs that enhancing the cellular SOD activity encouraged a radioprotective effect [9–13]. However, the drug delivery technologies of gene therapy were not comfortable for a human being. Wild SOD pretreatment was proved to be inefficient due to its inability of intracellular delivery. SOD mimics provide another potential development pattern of SOD. Mn porphyrins are most valid SOD mimics up to now and have been proven to be anticancer and radioprotective [30]. However, it still needs further improvement to match the catalytic efficiency of the natural enzyme. Otherwise, their action mechanism is varied due to vastly different sterics [30].

In our previous study, we gave SOD the capability of intracellular delivery with the fusion of cell-penetrating TAT-PTD. Both of monofunctional and bifunctional cell-permeable SOD, SOD-TAT, and GST-TAT-SOD can enter into cells freely [23]. With the additional fusion of another antioxidant enzyme GST, bifunctional GST-TAT-SOD was proved to be superior to monofunctional SOD-TAT and amifostine. It can remarkably clear up intracellular redundant ROS of irradiated normal cells, maintain their antioxidant system, enhance their colony-forming ability, and suppress apoptosis [23].

In this study, splenic GST activity of treated or untreated irradiated mice was not significantly different while the MDA level and SOD activity of the spleen were significantly increased and decreased by radiation, respectively. They were remarkably got down and elevated, respectively, when mice were given GST-TAT-SOD (Table 1). Therefore, the superior protective effect of GST-TAT-SOD on the irradiated spleen may be due to its powerful antioxidant capacity.

Otherwise, the present study denoted a significant ascent and reduction in liver MDA level and SOD activity, respectively, due to X-ray irradiation. Moreover, GST-TAT-SOD treatment before irradiation presented the superior ability to maintain the hepatic antioxidant system compared with amifostine pretreatment (Table 2). The changes in body weight, an indirect measure of gross physiology, also revealed that GST-TAT-SOD pretreatment imparted much better result than amifostine pretreatment although there was no significance between the treated and untreated mice exposed to irradiation (Figure 1). The result suggested that GST-TAT-SOD offers more comprehensive protection in irradiated mice. The subsequent survival study in mice exposed to irradiation further supported that hypothesis. All of the untreated mice died after a lethal acute dose of 8 Gy radiation on the 18th day postirradiation. Mortality of animal following radiation may be due to several factors like damages to the hematopoietic system and gastrointestinal system [31]. Forty percent of irradiated mice survived in the GST-TAT-SOD-administered group on the 18th day postirradiation. The survival percentage remained at 30% even after 30 days of postirradiation while that of the amifostine pretreatment group was 20% (Figure 5). The highest survival rate of the GST-TAT-SOD-administered group hinted that the protein would protect the gastrointestinal system of irradiated mice potentially.

Various chemical agents such as amifostine and other chemical compounds have been investigated as potential radioprotective drugs [32]. However, the inherent toxicity of these compounds warranted further search for safer and more efficient radioprotectors [6–8]. Our previous study verified the safety of GST-TAT-SOD on normal cells at a dose of 2000 U/ml [23]. The in vitro result is further confirmed in vivo. GST-TAT-SOD alone was observed not to induce any visible symptoms of toxicity during the whole observation period (Figures 2(), 3(), and 4(); Tables 1 and 2). Otherwise, GST-TAT-SOD could effectively transduce into different organs in mice such as the liver, lung, spleen, kidney, and brain by intraperitoneal injection [23]. However, amifostine cannot cross the blood-brain barrier which limits its application in radioprotection [33, 34]. Moreover, TAT is known to deliver proteins into cells and tissues in the form of the fusion protein by various routes, including oral administration [35] and parenteral administration such as transdermal administration [19, 20] and intraperitoneal injection [21, 36]. However, the most common administration of amifostine is subcutaneous administration and intravenous administration. All above results suggest that GST-TAT-SOD could be a promising therapeutic adjunct in radiation exposure as it is safe and effective and has broad biodistribution and various routes of administration. Although the present study offers experimental evidence only, its future potential for clinical relevance will warrant further attention.

## 5. Conclusions

In summary, the present study shows the radioprotective effects of the bifunctional GST-TAT-SOD on X-ray irradiation-induced damage in mice. It confirms that GST-TAT-SOD pretreatment is safe and superior to amifostine as a whole. It can effectively enhance splenic and hepatic antioxidant ability, the numbers of splenic white pulp, the thymus index, the spleen index, and the WBC of peripheral blood in irradiated mice which improved not only the living quality of irradiated mice but also the survival rates of mice receiving lethal radiation doses.

## Figures and Tables

**Table 1:** The effect of GST-TAT-SOD on the splenic antioxidant activity of irradiated mice.

	MDA (nmol/mg protein)	SOD activity (U/mg protein)	GST activity (U/mg protein)
CON	1.11 ± 0.75	44.87 ± 7.78	22.58 ± 7.38
GST-TAT-SOD	1.12 ± 0.34	42.49 ± 7.20	22.26 ± 6.20
XRT	2.69 ± 0.33^#^	36.78 ± 6.22^#^	22.54 ± 8.60
XRT + AMFT	1.74 ± 0.61^∗^	62.07 ± 10.02^∗^	23.84 ± 5.31
XRT + GST-TAT-SOD	1.91 ± 0.57^∗^	69.96 ± 16.41^∗^	23.07 ± 9.24

Animals were sacrificed 7 days after 6 Gy whole-body irradiation. Then, the spleen was collected and homogenized. The splenic antioxidant activity was determined spectrophotometrically using their corresponding diagnostic reagent kits according to the manufacturer's instructions. Values are expressed as means ± SD (*n* = 8; compared with the control group, ^#^*P* < 0.05, compared with XRT group, ^∗^*P* < 0.05).

**Table 2:** The effect of GST-TAT-SOD on the hepatic antioxidant activity of irradiated mice.

	MDA (nmol/mg protein)	SOD activity (U/mg protein)	GST activity (U/mg protein)
CON	0.95 ± 0.50	1112.52 ± 220.08	23.75 ± 2.52
GST-TAT-SOD	1.05 ± 0.32	954.22 ± 260.04	21.49 ± 6.96
XRT	1.90 ± 0.34^#^	840.47 ± 140.48^##^	20.50 ± 4.00
XRT + AMFT	0.91 ± 0.44^∗^	906.62 ± 168.83	21.82 ± 4.84
XRT + GST-TAT-SOD	0.71 ± 0.14^∗^	1123.21 ± 289.10^∗∗^	22.81 ± 8.27

Animals were sacrificed 7 days after 6 Gy whole-body irradiation. Then, the liver was collected and homogenized. The hepatic antioxidant activity was determined spectrophotometrically using their corresponding diagnostic reagent kits according to the manufacturer's instructions. Values are expressed as means ± SD (*n* = 8; compared with control group, ^#^*P* < 0.05, ^#^^#^*P* < 0.01, compared with XRT group, ^∗^*P* < 0.05, ^∗∗^*P* < 0.01).
